# Supplementary material for: Are carbon emissions trading and green financial instruments synergistic? -Comprehensive quantitative research based on content analysis
Source: PLoS One. 2024 Mar 7;19(3):e0298601. doi: 10.1371/journal.pone.0298601 (PMC10919601; doi:10.1371/journal.pone.0298601)
Supplement: S1 Data — (ZIP) [file pone.0298601.s001.zip › Data/地级市Z_F工作报告.zip!/╜¡╬≈/╥╦┤║╩╨/└┤╫╘╣1⁄2╓┌║┼í╛┬φ┐╦╩2╛▌═°í┐_2018.docx]

各位代表：

　　现在，我代表市人民政府，向大会报告工作，请予审议，并请各位政协委员和列席会议的同志提出意见。

　　一、2017年工作回顾

　　2017年是宜春发展进程中不寻常的一年。举世瞩目的党的十九大胜利召开，确立了习近平新时代中国特色社会主义思想的历史地位，开启了全面建设社会主义现代化国家新征程，标定了新时代宜春发展的方向和坐标。省委“创新引领、绿色崛起、担当实干、兴赣富民”工作方针的深入实施，充分激发了广大干部群众干事创业的热情，为宜春更好更快发展注入了强大动力。市委四届三次全会进一步厘清思路、明晰定位，凝聚了推动宜春新一轮大发展的共识共为。

　　一年来，全市上下认真贯彻落实党中央、国务院和省委、省政府及市委各项决策部署，紧紧围绕建设美丽宜春决胜全面小康奋斗目标，统筹做好稳增长、促改革、调结构、优生态、惠民生、保安全各项工作，经济社会保持平稳健康发展，完成了市四届人大二次会议确定的主要目标任务，实现了本届政府的良好开局。

　　——经济增长的良好势头让我们深受鼓舞。预计全市实现生产总值2030亿元、增长9%；财政总收入354.3亿元、增长10.4%，其中一般公共预算收入225.3亿元、同口径增长8.1%，税收收入占财政总收入比重达到83.3%，高出全省平均水平4.5个百分点；规模以上工业增加值增长9.2%；500万元以上固定资产投资2060亿元、增长13.3%；社会消费品零售总额670亿元、增长12.4%；实际利用外资7.74亿美元、增长9.4%；外贸出口167亿元、增长7.7%。财政总收入超20亿元县（市、区）达到8个。丰城、樟树实现全国县域经济与县域基本竞争力百强县位次前移。

　　——产业发展的阶段成果让我们倍感振奋。致力打造“亚洲锂都”、振兴“中国药都”。锂电新能源产业发展迅猛，“中国药都”振兴工程上升为省级战略，预计锂电新能源、医药产业实现主营业务收入260亿元、330亿元，分别增长62.1%、10.8%；“八大工业支柱产业”实现主营业务收入3680亿元。大力发展六大农业特色产业，预计农业总产值达到486.5亿元，肉牛出栏54.35万头，绿色食品原料基地、有机农产品基地、富硒农产品基地、高产油茶林、中药材种植面积分别达到191.2万亩、110.4万亩、25万亩、118万亩、31.6万亩。“宜春大米”获评国家农产品地理标志，“袁州茶油”成功注册中国地理标志。旅游服务业加快发展，接待游客7196.9万人次，实现旅游综合收入611.3亿元，分别增长35.8%和41.7%。明月山机场旅客吞吐量突破50万人次，创造了民航发展的“宜春速度”。

　　——民生改善的实际成效让我们由衷欣慰。预计城镇、农村居民人均可支配收入达到29813元、13756元，分别增长8.6%、8.8%。年初确定的10件民生实事全面办成，4.08万人脱贫、40个省定贫困村、13个市定贫困村退出，产业扶贫工程实现全覆盖；完成城市棚户区改造19804套，改造农村危房5592户，分配公共租赁住房7599套；发放“财政惠农信贷通”贷款14亿元；新建25户以上自然村通水泥路3500公里，改造农村公路危桥103座。全面推行“一次办好”政务便民服务，全省首家市级行政服务中心智能化大厅投入使用，12345政府服务热线平台即将开通。

　　一年来，主要做了以下工作：

　　（一）主攻产业升级，动能培育与品牌建设同步推进。大力实施产业兴市、工业强市战略，深入推进工业、农业、旅游服务业升级，三次产业比由上年的15.5:45.1:39.4调整为13.8:45.8:40.4。工业经济提质增效。新增“专精特新”中小企业65户、规模以上工业企业311户，工业用电量达到132亿千瓦时、增长16.8%，均居全省前列。新增高新技术企业104家，预计高新技术产业增加值增长11%；规模以上工业企业主营业务收入、利润总额分别增长16%、28%。成功举办了院士国医名师宜春行、第三届江西（宜春）锂电新能源产业合作推进会等系列活动。中国驰名商标、省著名商标总数均列全省第一。袁州机电产业基地获批中国锂电新能源产业园，宜春经开区国家锂电新能源高新技术产业化基地获评最佳投资环境锂电产业集群，丰城循环园区列为全国首批绿色园区，宜丰绿色高效储能系统制造和丰城高新园区装备制造产业入选省级重点工业产业集群，高安工业园晋升为省级高新技术产业园区。现代农业优势凸显。粮食总产达到85.6亿斤，粮食安全省长责任制考核成绩列全省第一，上高荣获全国绿色食品高产高效创建标兵县。“三品一标”认证数达到860个。市级以上农业产业化龙头企业达到397家，注册农民合作社6978家、家庭农场2056家。土地集约节约利用被国务院通报表扬，连续五年获评全省耕地保护成效显著设区市。宜春入选第二批国家农产品质量安全市创建试点单位，丰城列为国家农业可持续发展试验示范区，高安巴夫洛列入国家田园综合体试点。旅游服务业活力迸发。全面启动国家全域旅游示范区创建，新增国家4A级景区2个、5A级乡村旅游点1个。成功举办了第11届月亮文化旅游节，明月山获评国家级旅游度假区，温汤镇获评世界温泉健康名镇。预计服务业实现增加值820亿元，电子商务交易额达到429亿元、增长35.3%，新增A级物流企业10家。与华为公司签订战略合作协议共建云计算大数据中心，智慧经济产业特色小镇建设有序推进。

　　（二）狠抓功能提升，城乡面貌与人居环境同步改善。坚持规划引领，着力优化城乡环境。城乡布局更加合理。启动了城乡总体规划编制，制定了建设赣西地区文体教育中心、休闲旅游度假商贸中心、医疗健康养生中心实施规划，做大中心城区做强市本级的方向和路径进一步明晰。启动了宜万经济走廊规划编制，宜万快速通道即将开工。出台了加强农民建房规划管理实施意见。城乡功能不断完善。开展中心城区城市功能三年提升行动，明月山机场扩建、四方井水利枢纽工程、220国道绕城改建全面开工，市民体育运动中心建设正式启动，“一通道三天桥”投入使用，市人民医院北院开业运营。樟树阁山镇入选第二批全国特色小镇，5个镇入选全省首批特色小镇。龙头山水电站枢纽工程稳步实施。城乡环境明显改观。拆除城市建成区违法建设108.5万平方米，“三线六边”环境得到改善。狠抓中央环保督察反馈问题整改，开展了“四尘三烟三气”污染综合整治，加强了饮用水水源地保护和劣Ⅴ类水治理，空气质量、河流水质持续好转。落实《国家生态文明试验区（江西）实施方案》，启动了生态+大健康产业发展等5项试点。靖安入选首批国家生态文明建设示范县和 “绿水青山就是金山银山”实践创新基地，奉新实现省级生态乡镇全覆盖。“整洁美丽、和谐宜居”新农村建设加快，3424个村点“七改三网”全面完成。

　　（三）深化改革创新，内生动力与发展活力同步释放。坚持向改革开放要动力、向创新创业要活力，发展潜力不断增强。改革力度进一步加大。全面推行“二十九证合一”改革，新增市场主体5.3万户。关闭煤矿11处、退出产能60万吨，煤炭化解过剩产能工作走在全省前列。商品房库存去化时间下降到8.1个月。深入开展降成本优环境专项行动，累计为企业减负102亿元。推进相对集中行政许可权改革和深化综合行政执法体制改革试点，市行政审批局挂牌成立。大力推动“众创业、个升企、企入规、规转股、扶上市、育龙头、聚集群”，实体经济不断壮大。加强了市本级行政事业单位和企业国有资产清收统管整合，“1+5”投融资平台组建工作基本完成。农村土地确权登记颁证工作通过省级验收，铜鼓入选全国农村集体产权制度改革试点县。开放招商成效明显。组织参加赣京会、赣港会、赣台会等重大招商活动，分别在北京中关村、人民大会堂举办了新经济新技术产业对接会、绿色有机产品展示推介会等活动，引进省外2000万元以上项目330个、资金628亿元，其中亿元以上项目150个，科陆电子、宜联科技等12个项目投资均超10亿元。成功举办了第二届宜商大会暨首届宜春投资与发展论坛，新成立异地宜春商会5个。中国WTO烟花爆竹技术性贸易措施研究评议基地落户我市，宜春海关开关运营。创新创业更具活力。预计全社会研究与试验发展经费投入占GDP比重达到1.2%。新增国家级众创空间和星创天地6家、国家知识产权优势企业5家、省级以上创新平台9个、省级科技奖励8项，获评国家创新驱动示范市。全省首个航天育种院士工作站落户万载。发放创业担保贷款19亿元，直接扶持1.14万人创业，带动4.6万人就业。预计金融机构新增贷款346.5亿元，增长22.1%。江西（奉新）同和药业在深圳创业板上市，新增“新三板”挂牌企业2家。

　　（四）持续改善民生，民生福祉与公共服务同步增强。坚持财政支出向民生领域倾斜，全市民生支出342.5亿元、增长11.3%，占一般公共预算支出71.2%。社会保障得到加强。预计新增城镇就业5.8万人、转移农村劳动力8.4万人，城镇登记失业率控制在3%以内。城乡居民基本养老保险参保率提升至99.68%。全市医保信息系统接入全国异地就医结算平台。市残疾人康复托养中心全面竣工。社会事业协调发展。启动实施中心城区教育提升三年行动计划，新改扩建学校6所，新增校舍面积12万平方米。10个县（市、区）均通过全国义务教育发展基本均衡县认定。靖安入选全国义务教育教师队伍“县管校聘”管理体制改革示范区。电影《我的上高》在央视播出，并荣获百合奖优秀故事片一等奖，高安采茶戏《孙成打酒》入选文化部2017年度剧本扶持工程。累计入选“中国好人榜”166人，奉新支月英当选感动中国年度人物。启动了国家健康城市试点。成功举办全国农耕健身大赛和市第五届运动会。社会大局和谐稳定。深化法治宜春、平安宜春建设，公众安全感进一步提升。加强安全生产专项治理，安全生产事故起数和死亡人数实现“双下降”。分别成立了全省首支安全生产、食品药品和环境犯罪侦查支队。推进食品安全示范城市创建和“四小”行业治理，全省首张食品小作坊登记证在铜鼓颁发。全国地质灾害防治高标准“十有县”实现全覆盖。深入开展群访群治、集中整治“六大行动”，市信访局获评全国信访系统先进集体。

　　（五）加强自身建设，政府作风与行政效能同步提升。创新服务举措、提高服务质量，不断提升政府行政效能。法治观念明显增强。严格执行重大行政决策机制和政府法律顾问制度，市县两级公共法律服务中心全部建成并投入使用。加强督查工作，出台了重大项目巡查办法，建立了重大项目等工作推进微信群。认真办结人大代表建议、政协委员提案，满意和基本满意率达到100%。全面落实《政府信息公开条例》。政务环境持续优化。扎实推进“两学一做”学习教育常态化制度化，积极开展作风突出问题集中整治，干部作风进一步好转。全面推行“双随机、一公开”监管，行政审批办理时限压缩67%以上。完善了权责清单制度体系，乡镇政府政务服务事项清单对外公布。廉政建设深入推进。认真贯彻落实中央八项规定精神、国务院“约法三章”和省委、省政府有关规定，加强“三公”经费和政府债务管理，严格执行政府集中采购、项目招投标、土地招拍挂等制度，强化政府公共工程和专项资金使用审计。落实“一岗双责”，严肃查处部门和行业不正之风。

　　与此同时，国防动员和后备力量建设、人民防空、拥军优属、国家安全、民族宗教、外事侨务、妇女儿童等工作继续加强；邮政通信、石油、烟草、气象、水文、盐业、无线电管理、防震减灾、统计、档案、史志和红十字会等工作取得新进展。

　　各位代表，过去的一年，我们收获了不少荣誉，取得了来之不易的成绩，这是省委、省政府和市委正确领导、科学决策的结果，是市人大、市政协和社会各界有效监督、大力支持的结果，是全市上下风雨同舟、奋力拼搏的结果。在此，我代表市人民政府，向全市人民，向各位人大代表、政协委员和离退休老同志，向各民主党派、工商联、人民团体，向驻宜部队官兵、公安干警，向所有关心、支持宜春发展的各界人士，表示衷心的感谢并致以崇高的敬意！

　　在看到成绩的同时，我们深知，全市经济社会发展还存在不少困难和问题，政府工作还有不少不尽人意的地方，主要是：经济总量偏小，产业层次不高，新的经济增长点有待加速培育；科技创新能力不强，科技成果转化率还比较低；中心城区辐射带动力偏弱，县域发展不平衡，统筹城乡发展的任务仍然艰巨；公共服务资源配置不够均衡，民生短板较多，养老、医疗、教育等方面还存在不少薄弱环节；政府职能转变步伐还需进一步加快，依法行政能力有待提高，等等。对此，我们将立足当前，着眼长远，认真对待，着力解决，决不辜负全市人民的信任与重托。

　　二、2018年主要工作

　　2018年是贯彻党的十九大精神的开局之年，是全面落实市委四届三次全会精神的关键之年，做好全年各项工作意义重大。我们要紧贴新时代，把握和运用好新思想、新战略、新举措，解决好宜春新一轮大发展面临的新矛盾、新挑战、新问题，不断提高经济发展的质量和效益，实现更好更快发展。

　　今年政府工作的总体要求是：全面贯彻党的十九大、中央经济工作会议、省委十四届五次全会和市委四届四次全会精神，以习近平新时代中国特色社会主义思想为指导，深入落实省委“创新引领、绿色崛起、担当实干、兴赣富民”工作方针，坚持稳中求进工作总基调，贯彻新发展理念，按照高质量发展的要求，大力实施产业兴市、工业强市战略，围绕把宜春打造成赣西区域性中心城市，把中心城区打造成“三个中心、一个基地”，把县市打造成个性彰显、活力十足、竞相发展的特色县域经济板块，统筹城乡发展，深化改革开放，加快生态文明建设，持续增进民生福祉，着力打好防范化解重大风险、精准脱贫、污染防治三大攻坚战，推动经济社会持续健康发展，奋力迈出建设美丽宜春决胜全面小康的坚实步伐，努力谱写新时代中国特色社会主义的宜春篇章。

　　今年全市经济社会发展主要预期目标是：生产总值增长8.8%左右；财政总收入增长8%左右，其中一般公共预算收入增长8%左右；规模以上工业增加值增长9%左右；500万元以上固定资产投资增长11.5%左右；社会消费品零售总额增长12.4%左右；实际利用外资增长9%左右；外贸出口增长3%左右；城镇居民人均可支配收入增长8.5%左右；农村居民人均可支配收入增长9%左右；居民消费价格总水平涨幅控制在3%左右；城镇登记失业率控制在4.5%以内；节能减排完成省下达的计划任务。在实际工作中，要力争更好的结果。

　　实现上述目标和要求，重点要抓好以下六个方面：

　　（一）贯彻新理念，大力振兴实体经济

　　实体经济是建设现代化经济体系的核心领域。要围绕实现“四年翻一番决战工业八千亿”目标，深入实施“8647”主攻计划，重点培育新兴产业，整体提升传统产业，着力构建现代产业体系。

　　壮大产业规模。坚持做大总量、做优结构，重点在延链、补链、壮链上下功夫。围绕打造“亚洲锂都”、振兴“中国药都”，加强锂矿资源整合，拓展中医药产业空间布局，加快发展锂电新能源和医药产业，力争主营业务收入分别达到350亿元以上、480亿元以上。加强新兴产业培育，大力发展电子信息、先进装备制造产业，力争主营业务收入分别达到550亿元以上、430亿元以上。加快传统产业升级，重点围绕建材、食品、纺织、化工等产业，开展“互联网+”、“品牌+”和“标准化+”改造行动，提升产品、技术、工艺和装备的现代化水平。加速重点产业集聚，以特色产业基地、标杆企业、重点项目为抓手，实行“一产一策”、“一链一策”、“一企一策”，打造一批特色鲜明、关联配套、竞争力强的产业集群，力争全市省级重点工业产业集群主营业务收入达到2800亿元以上。发展总部经济，鼓励企业在我市设立总部。

　　培育骨干企业。引导企业通过战略合作、兼并重组、技改扩能等方式，推动优势资源集聚。支持中小企业走“专精特新”之路，打造一批专业化“小巨人”企业。做好“入规纳统”工作，力争新增规模以上工业企业200家。建立工业项目定期调度机制，力争100个亿元以上工业项目竣工投产，完成工业投资1500亿元以上。设立规模700亿元的发展升级引导基金，扶持实体企业发展壮大。实施企业管理提升计划，推进对标贯标工作，促进企业提质增效。

　　做实平台支撑。坚持聚焦主业、做强产业，打造先进制造业、高新技术产业和生产性服务业集聚区，力争工业园区主营业务收入突破5000亿元。抓好200万平方米以上的标准厂房建设，加强低效闲置工业用地“二次开发”，完善生产生活综合配套，建设现代工业新城。支持宜春经开区做大做强，力争实现主营业务收入450亿元以上。探索建立宜春经开区与有关县（市、区）合作机制，发展协同共建、利益共享的“飞地经济”。加快省级锂电产品监督检验中心建设，同步创新中国成立家锂电产品监督检验中心。

　　（二）找准新坐标，加快推动区域协调发展

　　区域协调是解决发展不平衡不充分的根本途径。要围绕把宜春打造成赣西区域性中心城市，加大投入、完善功能，强化支撑、均衡发力，促进城乡协同发展、共同繁荣。

　　推动“四区”融合。统筹城市空间布局，按照“多规合一”方式，完成全市城乡总体规划编制。加快教体新区建设，支持宜春职业技术学院异地搬迁、宜春学院建设宜春大学。按照全域旅游理念，以明月山旅游景区为龙头，深入挖掘禅宗文化、月亮文化内涵，抓好首期25条、502.9公里旅游公路建设，进一步做响宜春旅游品牌；支持靖安三爪仑创新中国成立家5A级旅游景区、温汤创新中国成立家温泉旅游名镇，加快建设宋城·明月千古情项目。全面开展国家健康城市试点，启动市人民医院北院二期住院大楼建设，组建市本级医疗集团和市中医院，实施“互联网+智慧医疗”工程，促进医疗卫生事业与大健康产业融合发展。抓紧理顺市本级和“四区”的关系，建立完善与事权、财权相匹配的体制机制。按照“能减就减，能放都放”的原则，持续加大简政放权力度，支持各地发挥优势，各展所长，错位发展，构筑县域经济发展新优势。推进宜春经开区产城融合，打造宜万经济走廊，拉动辐射上高、宜丰、铜鼓、万载加快发展。支持丰城、樟树、高安、靖安、奉新主动对接南昌，实现融合发展。

　　开展“城市双修”。加强环境综合治理，一条一条道路整治、一片一片区域治理、一个一个街道美化，达到“串点、联线、成片”的效果。按照“缺什么、补什么”的原则，中心城区实施基础设施、功能提升、文体设施、环境改善、经济发展5大类22项民生工程。推进海绵城市和城乡农贸市场标准化建设。谋划咸宜井（吉）铁路、铜鼓至通城高速公路项目，抓好南昌经靖安和奉新至修水城际铁路、宜春经井冈山至遂川高速公路项目前期工作，启动实施大塘片区路网配套、飞剑潭备用水源、锦绣大道改造等工程，加快建设四方井水利枢纽、地下综合管廊、铜万高速公路宜丰联络线等项目。

　　加速乡村振兴。坚持农业农村优先发展，以“六调”为方向，加快推进农业农村现代化，力争农业总产值突破500亿元。落实粮食安全责任制，确保粮食总产稳定在85亿斤以上。培育壮大六大特色产业，力争肉牛出栏65万头，绿色食品原料基地、有机农产品基地、富硒农产品基地、高产油茶林、中药材种植面积分别达到245万亩、120万亩、32万亩、130万亩、40万亩。聚力打造农产品品牌，力争成功创建地理标志证明商标2件、地理标志保护品牌1个，实现全国百强农产品区域公用品牌零的突破。加强农产品质量安全监管，争创国家农产品质量安全市。支持铜鼓发展全域有机农业。按照“田成方、渠相通、路相连、涝能排、旱能灌”的标准，建设49万亩高标准农田。大力发展功能农业、智慧农业、设施农业、特色农业，推动丰城生态硒谷、高安巴夫洛、万载恒晖、奉新万亩生态猕猴桃等现代农业园扩容提质。建立动态评估制度，加快7个首批市级特色小镇建设。深入推进“整洁美丽、和谐宜居”新农村建设，抓好人居环境整治，规范农村建房秩序，实施“厕所革命”，打造美丽示范风景线。加强农村精神文明建设，树立文明乡风。

　　（三）培育新动能，全面深化改革开放

　　改革开放是破解发展难题的金钥匙，是厚植发展优势的着力点。要坚定不移深化改革、扩大开放，深入实施创新驱动战略，为经济社会发展注入强大动力。

　　全面深化改革。深化供给侧结构性改革，落实“三去一降一补”。深入实施国资国企改革，建立健全“1+N”制度框架，按照现代化企业制度管理要求，推进“1+5”投融资平台向实体型企业转化，放大融资能力，降低融资成本。深化“放管服”改革，突出抓好“多证合一”登记制度改革、相对集中行政许可权改革试点、综合行政执法体制改革试点，确保市行政审批局正常运行；创新政府配置资源方式，促进公共资源配置高效、公平、可持续；开展政府绩效管理考核，进一步提高行政效能；启动检验检疫职能延伸至县（市、区）改革试点。深化农村土地制度改革，完善“三权”分置制度，统筹抓好农村集体产权制度、宅基地改革试点和林权、水权、农垦、供销合作社等改革工作。深化教育文化、医药卫生、社会保障、收入分配等领域改革。

　　扩大对外开放。加大招商引资力度，精心组织专题活动，力争引进亿元以上项目180个、10亿元以上项目20个。落实重大项目管理办法，促进高安中兴通讯智慧城市、袁州宝科精密钟表产业园、上高杭萧钢构、万载三一重工装配式建筑、宜丰科士达等项目落地，加快袁州宜联科技二期、丰城科陆电子、樟树岐黄健康小镇、高安通瑞新能源、上高新威动力扩建、万载南氏锂电二期、奉新璞泰来新材料、宜春经开区远东福斯特二期、科陆储能等项目建设。主动融入“一带一路”、长江经济带等国家战略，不断提升双向开放水平。启动“宜春港”建设，推进万载公路口岸、樟树水运口岸、高安铁路口岸查验区后续建设，争取在宜春开通“赣欧班列”以及至宁波或深圳快速班列，打造赣西口岸节点。开展宜春航空物流园前期工作。大力实施“宜商回归工程”，加快宜商网络建设，探索建设市级宜商回归创业园。积极鼓励企业开展自营出口，提高生产型企业出口比重，调优对外贸易结构。抓好WTO烟花爆竹技术性贸易措施研究评议基地建设。

　　推进科技创新。紧紧抓住科技创新这个“牛鼻子”，推动经济发展由要素驱动向创新驱动转变。加大科技研发投入，力争全社会研究与试验发展经费投入占GDP比重达到1.45%。狠抓关键技术攻关，提高创新供给能力，力争新增高新技术企业100家，高新技术产业增加值达到340亿元以上，科技进步贡献率提高到58%。强化创新平台支撑，力争建成全产业链研究机构1—2个，获批国家级众创空间和星创天地2个、省级创新平台6个。支持宜春丰城高新技术产业园区创新中国成立家级高新区，争创省级高新技术园区1—2个。加快科技成果转移转化，力争实施产学研合作项目210项以上。加强知识产权保护，力争专利申请量增长25%以上。推进大众创业、万众创新，开展“双返双创”活动，实施“百千万人才引进工程”、“领军型创新创业团队引进培育计划”，力争新增省级以上创新人才和团队3个，院士工作站1—2个，博士后科研工作站2—3个。

　　发展新经济。大力发展以大数据产业为代表的新经济，建设以产业为支撑的智慧经济产业特色小镇，培育壮大新动能。抓好云计算大数据中心永久机房建设，加强“云上宜春”示范应用及互联网、大数据、人工智能与重点产业深度融合创新，延伸大数据、云计算产业链，力争引进大数据企业30家以上。建设工业云平台，推进两化融合和智能制造试点示范，打造一批“数字化车间”、“智能工厂”、“智能制造基地”，形成一批“互联网+”示范企业和示范园区。发展现代物流、现代金融、电子商务、工业设计、健康养老等现代服务业，促进大数据、云计算、物联网等新技术深度融入教育、文化、医疗、金融等领域，形成更多新的服务业态和商业模式。培育文化创意产业，争创国家级文化产业基地1—2个。积极对接多层次资本市场，力争引进外埠银行1家，新增境内外上市企业1家、“新三板”挂牌企业5家。

　　（四）彰显新优势，扎实推进生态文明建设

　　建设生态文明是实现永续发展的千年大计。要倍加珍惜绿色生态这一最大财富、最大优势、最大品牌，推动生态文明共建共享，构建绿色发展新格局。

　　狠抓环境治理。全力推进中央环保督察和省环保督察反馈意见整改，补齐生态环境保护短板。抓好污染源普查，建立健全重点污染源档案、污染源信息数据库和环境统计平台。深入开展“四尘三烟三气”污染综合整治，不断提升城市和工业治理水平，切实改善环境空气质量。全面落实“河长制”责任体系，加强饮用水水源保护，整治城市黑臭水体，坚决消灭劣Ｖ类水。抓好土壤污染状况详查采样、监测工作，出台土壤污染治理修复规划，规范危险废弃物处置，严防土壤污染。提高畜禽养殖废弃物综合利用水平，减少农药、化肥使用量，降低农业面源污染风险。

　　筑牢生态屏障。用好用活昌铜高速生态经济带发展基金，打造昌铜百里生态风光带。加强山水林田湖草系统保护与综合治理，构建生态廊道和生物多样性保护网络，全面提升自然生态系统的稳定性和生态服务功能。支持靖安生态云、奉新生态+、宜丰天然阔叶林保护、铜鼓非国有森林赎买等试点工作。实施森林质量提升工程，完成人工造林14.2万亩、封山育林14.4万亩、退化林修复19.1万亩、森林抚育45.6万亩。强化水土流失综合治理，推进耕地休养生息试点。落实国土空间用途管制要求，严守生态保护红线、永久基本农田、城镇开发边界三条控制线。开展国家生态文明建设示范县和省级生态县创建，抓好省级生态文明示范县和示范基地建设。加强节能工作，打造一批绿色机关、绿色家庭、绿色学校和绿色社区。

　　健全制度体系。对照《国家生态文明试验区（江西）实施方案》，重点抓好我市5大改革试点任务，加快形成一批可复制可推广的“宜春经验”。建立资源环境承载监测预警机制，严格实行重点生态功能区产业准入负面清单制度。实施清洁能源替代工程，推进排污权、碳排放权、用能权等交易试点。加大生态保护补偿力度，健全重点领域生态保护补偿机制。加强生态文明建设目标评价考核，开展领导干部自然资源资产离任审计，落实党政领导干部生态环境损害责任追究实施细则。

　　（五）顺应新期待，着力增进民生福祉

　　民之所望，施政所向。坚持把保障改善民生作为政府工作的出发点和落脚点，时刻把老百姓的安危冷暖放在心上，集中力量办好20项民生实事，不断增强人民群众的获得感和幸福感。

　　聚力脱贫攻坚。坚持“两不愁、三保障”标准，统筹力量，加快55个深度贫困村脱贫摘帽。深入实施产业扶贫全覆盖、健康扶贫再提升、易地扶贫搬迁再精准、教育扶贫再对接、贫困村庄整治再推进五大工程，确保3.67万建档立卡贫困人口脱贫、39个省定贫困村和7个市定贫困村退出。探索推进“合作社＋基地＋农户”模式，推动贫困村发展“一村一品”特色产业。层层压实责任，完善专项扶贫、行业扶贫、社会扶贫“三位一体”大扶贫格局，强化督察考核和责任追究，确保脱真贫、真脱贫。

　　提升公共服务。扩大职业技能培训规模，健全市县公共就业网和“两微一端”平台，力争新增城镇就业4.72万人。统筹城乡义务教育，大力发展公办和普惠性民办学前教育，力争80%以上的乡镇建有公办中心幼儿园。深入实施中心城区教育提升三年行动计划，加快教育信息化和教育工程建设。推进“数字档案馆”建设，实现档案信息资源互联互通。发展居家社区养老服务，促进医养融合，打造具有宜春特色的养老服务品牌。做好全国基层中医药工作先进市复审迎检工作。完善公共法律服务体系，建立线上线下法律服务网络。启动国家文明城市创建。抓好江西七O三电视台新台建设和基层综合文化服务中心新改扩建项目，推进基本公共文化服务标准化、均等化。加强社区服务平台建设，提升服务群众的能力和水平。

　　加强社会保障。按照兜底线、织密网、建机制的要求，加快建立覆盖全民、城乡统筹、权责清晰、保障适度、可持续的多层次社会保障体系。实施全民参保计划，扩大社会保险覆盖面，促进保险行业健康发展。以“多险合一”系统上线为契机，加大“互联网+社会保险”便民服务力度。推进住房制度改革和长效机制建设，加快城市棚户区和农村危房改造步伐，让更多的困难群众住有所居。高度关注城市弱势群体，加大社会救助保障力度，完善特困人员救助供养制度。加强计划生育目标管理，优化生育保健服务。扎实开展农村留守儿童健康关爱工作。

　　维护和谐稳定。全面推进安全生产领域改革发展，落实“党政同责、一岗双责、齐抓共管、失职追责”和“三管三必须”要求，督促企业严格履行安全生产主体责任。狠抓科技兴安，开展“机械化换人，自动化减人”专项行动。严格安全监管执法，深入开展安全生产隐患排查治理和重点行业领域专项整治，有效防范和遏制各类安全事故发生。加强应急体系建设，启动市级救灾应急物资储备库建设，加快温汤赣西人防疏散基地建设，提升防灾减灾救灾能力。实施食品安全战略，全域推进食品安全示范城市创建。巩固平安宜春、法治宜春建设成果，健全立体化、信息化社会治安防控体系，抓好金融领域风险防范工作，依法打击各类违法犯罪行为。开展阳光信访、责任信访、法治信访，妥善解决群众合理合法诉求。

　　（六）担当新使命，努力建设人民满意政府

　　站在新起点，面对新形势，我们要认真践行“担当为要、实干为本、发展为重、奋斗为荣”的为政理念，进一步加强自身建设，切实增强工作本领，着力塑造政府新形象。

　　讲政治，提高执行力。牢固树立 “四个意识”，坚决维护以习近平同志为核心的党中央权威和集中统一领导，坚决维护习近平总书记在党中央、在全党的核心地位。自觉用习近平新时代中国特色社会主义思想武装头脑，坚定理想信念，牢记党的宗旨，提高政治能力。顾大局、讲团结，始终保持工作一盘棋、干事一条心、发展一个调，齐心协力抓好党中央、国务院和省委、省政府及市委各项决策部署的落实落地，确保政令畅通、令行禁止。

　　敢担当，发挥创造力。心底无私天地宽。彰显 “功成不必在我、功成必定有我”的胸怀和境界，不贪一时之功，不图一时之名。倡导敢想敢干、敢闯敢试、敢为人先的创新精神，敢于把宜春发展放到全省乃至全国范围内来审视和谋划，提高站位，拉升标杆，全心投入，力争各项工作走在全省前列。大兴调查研究之风，崇尚实干，力戒空谈，凡是定了的事，就要积极探索试着干，创新思路比着干。认真开展“不忘初心、牢记使命”主题教育，强化责任落实，健全激励机制和容错纠错机制，努力形成人人有责、人人担责、人人尽责的干事创业氛围。

　　重实效，强化操作力。抓工作贵在操作、重在实效。要学会十个指头弹钢琴，避免“眉毛胡子一把抓”，特别是对于一些业务性、操作性强的工作，要充分听取专家和社会各方的意见，让懂业务的人来谋划，让善操作的人来操作，让专业的人去考虑专业的问题、做专业的事。凡是重大决策和重要事项，都要认真研究、反复推敲，确保政府决策科学合理，避免走弯路。坚持问题导向，着力解决阻碍发展的瓶颈制约、事关群众利益的突出问题。发扬“钉钉子”精神，一个一个问题求突破，一项一项工作去推进，一件一件事情抓落实。

　　守底线，增强免疫力。打铁必须自身硬。要时刻保持清醒头脑，慎用权力这把“双刃剑”。增强法治观念，树立法治思维，做到依法履职、规范用权。自觉接受市人大依法监督和政协民主监督，主动接受社会监督和舆论监督，确保政府权力在阳光下运行。认真落实习近平总书记关于进一步纠正“四风”、加强作风建设的重要批示，严格遵守中央八项规定精神，切实履行“一岗双责”，驰而不息纠“四风”，坚决防止不良风气反弹回潮。加强财政监管和审计监督，严肃查处侵害群众利益的不正之风，着力打造清正清廉清明政府。

　　各位代表，迈步新时代，开启新征程，加快发展时不我待，全面小康重任在肩。我们要紧密团结在以习近平同志为核心的党中央周围，在省委、省政府和市委的坚强领导下，凝聚610万宜春人民的智慧和力量，埋头苦干，扎实工作，开拓进取，狠抓落实，为建设美丽宜春决胜全面小康、谱写新时代中国特色社会主义的宜春篇章不懈奋斗！
